# The Poggendorff illusion affects manual pointing as well as perceptual judgements

**DOI:** 10.1016/j.neuropsychologia.2009.07.024

**Published:** 2009-12

**Authors:** Dean R. Melmoth, Marc S. Tibber, Simon Grant, Michael J. Morgan

**Affiliations:** Henry Wellcome Laboratories, Department of Optometry and Visual Science, City University, Northampton Square, London EC1 V 0HB, United Kingdom

**Keywords:** Action, Perception, Dorsal, Ventral, Pointing, Illusions

## Abstract

Pointing movements made to a target defined by the imaginary intersection of a pointer with a distant landing line were examined in healthy human observers in order to determine whether such motor responses are susceptible to the Poggendorff effect. In this well-known geometric illusion observers make systematic extrapolation errors when the pointer abuts a second line (the inducer). The kinematics of extrapolation movements, in which no explicit target was present, where similar to those made in response to a rapid-onset (explicit) dot target. The results unambiguously demonstrate that motor (pointing) responses are susceptible to the illusion. In fact, raw motor biases were *greater* than for perceptual responses: in the absence of an inducer (and hence also the acute angle of the Poggendorff stimulus) perceptual responses were near-veridical, whilst motor responses retained a bias. Therefore, the full Poggendorff stimulus contained two biases: one mediated by the acute angle formed between the oblique pointer and the inducing line (the classic Poggendorff effect), which affected both motor and perceptual responses equally, and another bias, which was independent of the inducer and primarily affected motor responses. We conjecture that this additional motor bias is associated with an undershoot in the unknown direction of movement and provide evidence to justify this claim. In conclusion, both manual pointing and perceptual judgements are susceptible to the well-known Poggendorff effect, supporting the notion of a unitary representation of space for action and perception or else an early locus for the effect, prior to the divergence of processing streams.

## 1 Introduction

The two-stream hypothesis of visual perception (Goodale & Milner, 1992; Goodale, Jakobson, & Keillor, 1994; Hu & Goodale, 2000; Milner & Goodale, 1995) suggests that certain illusions will not affect motor responses since they are mediated by the dorsal stream, which operates via egocentric (absolute) metrics. In contrast, the allocentric (relative) metrics used for perception by the ventral stream are systematically biased by such illusions. Experimental studies have revealed dissociations consistent with this hypothesis, whereby subjects make accurate motor responses to illusory stimuli despite perceptual responses that are biased in accordance with the illusion (Aglioti, DeSouza, & Goodale, 1995; Haffenden & Goodale, 1998; Haffenden, Schiff, & Goodale, 2001; Mack, Heuer, Villardi, & Chambers, 1985; Servos, Carnahan, & Fedwick, 2000; Westwood, Chapman, & Roy, 2000; Westwood, Heath, & Roy, 2000). However, there are also reports of motor and perceptual responses being affected approximately equally by visual illusions (Daprati & Gentilucci, 1997; de Grave, Brenner, & Smeets, 2004; Elliott & Lee, 1995; Franz, 2003; Franz, Bülthoff, & Fahle, 2003; Franz, Fahle, Bulthoff, & Gegenfurtner, 2001; Gentilucci, Chieffi, Deprati, Saetti, & Toni, 1996; Meegan et al., 2004; Predebon, 2004; van Donkelaar, 1999), which have been used to argue in favour of a single representation of space for perceptual and motor responses (Franz et al., 2003).

One explanation for the latter findings is that the illusions used may generate biases early in the visual system, before the ventral and dorsal stream split, so that they are inherited by both streams (Milner & Dyde, 2003). Dyde and Milner (2002) explored this possibility by setting two illusions against each other to null overall bias. The simultaneous tilt illusion operates over short distances and is presumed to arise early in the visual system, perhaps from inhibitory connections between cortical columns in V1. In contrast, the rod-and-frame tilt illusion operates over much greater distances, thus putatively originating from higher visual cortical areas—presumably in the ventral stream. As a result, the simultaneous tilt illusion is inherited by both ventral and dorsal streams affecting both perceptual and motor responses, whilst the rod-and-frame illusion only affects perceptual responses. Consequently, when these two illusions were combined – by placing a tilted frame around a simultaneous tilt illusion stimulus – and set in opposite directions, the net effect on perceptual responses was nulled whilst motor responses remained biased in the direction specified by the simultaneous tilt illusion.

With notable exceptions, such as the above experiment, studies exploring potential dissociations between motor and perceptual biases have drawn heavily upon a small range of illusions. Most commonly used are the Müller–Lyer illusion, and size-contrast illusions such as the Ebbinghaus/Titchener. Other types of illusion are greatly under-represented in the literature. For example, the Poggendorff effect is a robust illusion of extrapolation misalignment quite distinct from size-contrast illusions. Despite extensive exploration of its perceptual effect, the only study we are aware of that compared this with motor responses reported an approximately equal impact of the Poggendorff illusion upon both response modes (Predebon, 2004). However, the motor response in this experiment was not a natural ballistic pointing movement but a form of manual estimation, whereby subjects gripped a rod which was positioned below the stimulus and slid it along a track to indicate their response. Such non-ballistic manual estimation tasks may actually be mediated by perceptual mechanisms (Franz, 2003; Haffenden & Goodale, 1998), and are unlikely to engage real-time visuo-motor dorsal stream processing. We wished to study responses to a Poggendorff figure using a rapid naturalistic pointing movement, conditions that are most suited to engaging dorsal stream mechanisms.

## 2 Materials and methods

### 2.1 Subjects

In Experiment 1 7 subjects with a median age of 28.2 years participated: 4 were the authors, 3 were naïve subjects. For Experiments 2–4 9 subjects with a median age of 24.3 participated: 4 were the authors, 5 were naïve subjects different to those from Experiment 1. All subjects were right-handed and had normal or corrected-to-normal vision. Informed written consent was obtained prior to inclusion and procedures were in accordance with the Declaration of Helsinki.

### 2.2 Stimuli

**Fig. 1:**
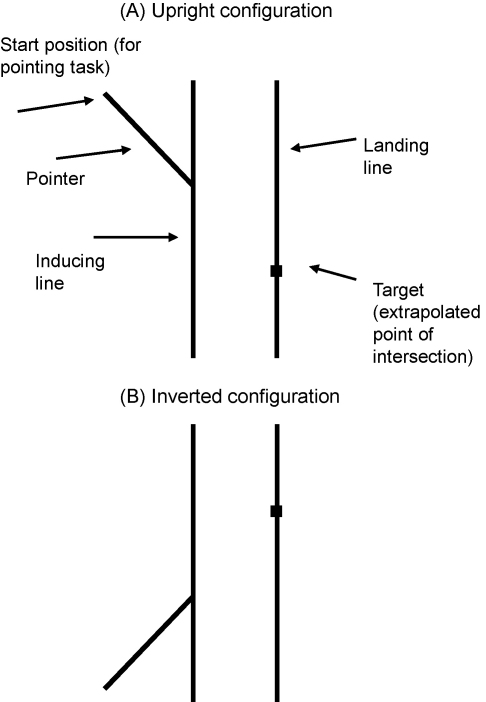
Experimental stimulus in (a) upright and (b) inverted configurations. The black square shows the true point of intersection if the oblique pointer is extrapolated to meet the vertical landing line. The Poggendorff effect creates the illusion that the extrapolated intersection is too low. By presenting both upright and inverted stimuli and noting the difference between the two, it is possible to observe the net effect attributable to the illusion, irrespective of any inherent cross-condition bias (e.g., a general tendency to make lower settings irrespective of the stimulus configuration).

Stimuli were presented on a vertically oriented Protouch 17-inch TFT flat-screen display (928 × 799 pixels; 60 Hz), via a PC fitted with a VSG graphics card (Cambridge Research Systems Ltd., Rochester, UK) running custom-written scripts for MATLAB (MathWorks Ltd., Cambridge, UK). On-screen pixel size was 0.36 mm and average background luminance was 55 cd/m^2^, whilst average luminance of the stimulus components was 130 cd/m^2^. Inducing and landing lines measured 25.4 cm × 0.07 cm with a 7.3 cm separation in the parallel conditions. Subjects were seated at a comfortable reaching distance from the screen (approximately 50 cm), so that the above stimulus dimensions in degrees of visual angle were approximately 28.5°, 0.08° and 8.4°, respectively. The oblique pointer was 5.9° in length and angled at −45° or +45° relative to the horizontal for top-down or bottom-up reaching conditions, respectively. See Fig. 1 for details. In Experiment 1 a randomised angular jitter in the range of ±5° was added to prevent stereotyped responses. Having confirmed that this made no difference to performance (Experiment 2) the jitter was not applied for the remainder of the experiments. For all experiments the on-screen position of the entire stimulus configuration was spatially jittered from trial-to-trial.

### 2.3 Procedure

For perceptual tasks, subjects initiated the measurement via a key press, which triggered stimulus presentation. A small 8 × 8 pixel marker was positioned randomly on the landing line below its true intersection with the extrapolated oblique pointer. By a method of adjustment subjects used the keyboard to move this on-screen marker to where they believed the intersection to be, at which point they pressed another key to log their response. The marker was then randomly re-positioned – this time *above* the true point of intersection – and another measurement was taken. The average of these two measurements was taken as the response for a particular trial so that each measurement was derived from two responses (one starting from below and one from above the point of true intersection). Stimuli were presented randomly either upright or inverted (50% of trials each) and either with or without the inducing line present (50% of trials each) with 5 repeats of each condition, giving a total of 40 responses. Responses were not time-constrained.

For the motor task a small lightweight infra-red reflective marker was attached to the nail of the right index finger. At the beginning of each trial a small 8 × 8 pixel square representing the start position was displayed either in the top-left or the bottom-left of the screen, and subjects placed their fingertip on it. Its position was jittered from trial-to-trial, but it always marked the origin of the as yet unseen oblique pointer line. When the experimenter initiated the presentation, the stimulus appeared and subjects had to make a rapid ballistic movement, pointing to the extrapolated intersection of the pointer line and the landing line. For Experiment 1 (‘No preview’), subjects were instructed to initiate a movement as soon as the stimulus appeared and the angle of the pointer was jittered randomly in the range ±0.5° to prevent stereotyped movements or pre-planning of trajectories. For Experiments 2–4 subjects were given a 3 s preview of the pointer (but not of the landing line) before they began their movement and the angle of the pointer was fixed: +45° and −45° for upright and inverted configurations, respectively. The cue to begin the pointing movement was the appearance of the landing line. The latter remained on for 750 ms, which provided enough time for subjects to complete their programmed movement, but meant the stimulus had disappeared before they could look back to the pointer to evaluate or adjust their finger position. Full vision of the hand and stimulus was allowed and thus the movement was made under visual guidance (closed-loop). However, no feedback was given regarding accuracy of responses.

For calibration purposes, on half of trials the true point of intersection was explicitly shown with an 8 × 8 pixel target on the landing line, providing an explicit target for the observers to point to. Responses in trials without the explicit target (i.e. when subjects were forced to perform an extrapolation) were measured relative to the average pointing position with the explicit target in the corresponding condition. Again, stimuli were presented either upright or inverted. Thus, with both orientations, with and without the point of intersection marked, with and without the inducing line present and with 5 repeats for each condition, a total of 40 measurements were taken. Movements were recorded using Qualysis ProReflex (Sweden) motion capture cameras, with a resolution of <0.4 mm.

Subjects’ responses (on-screen cursor position for the perceptual task or fingertip position for the motor task) were converted to angular errors, which were defined relative to the true trajectory from start position to extrapolated intersection: −45° and +45° (plus jitter) for the upright and inverted configuration, respectively. A positive angular error was defined as a bias in the direction predicted from angular expansion of the acute Poggendorff angle. To overcome any inherent response bias to a particular stimulus orientation, stimuli were presented both upright and inverted, and the angular bias to the inverted configuration was added to that for the upright configuration as the “net inversion effect”.

## 3 Results

### 3.1 Experiment 1: effects of the Poggendorff illusion on perceptual and pointing responses

**Fig. 2:**
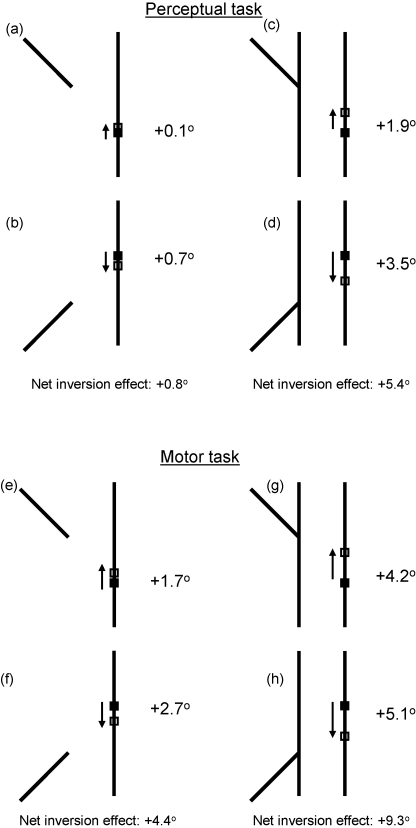
Results of Experiment 1, pictorial representations of the stimuli are shown for each condition along with the true extrapolated intersection of the pointer and landing line (filled squares) and the mean subject response (unfilled squares). Distances are not to scale. (a, b, e, and f) show upright and inverted stimulus configuration for the ‘pointer-and-landing-line-only’ (baseline) conditions for perceptual and motor tasks, respectively. (c, d, g, and h) show upright and inverted stimulus configurations of the full Poggendorff stimulus for perceptual and motor (pointing) response modes, respectively.

Fig. 2a–d shows that subjects succumbed perceptually to the Poggendorff illusion.

Thus, whilst perceptual judgements were largely veridical in the absence of an inducer (Fig. 2a and b: net inversion effect of +0.8°), observers were heavily biased in the direction of the classic Poggendorff effect when an inducer was present (Fig. 2c and d: net inversion effect of +5.4°). The Poggendorff effect was also manifested in the pointing responses, with a net inversion effect of +9.3° in the inducer present condition (Fig. 2g and h), compared to a baseline net inversion effect of +4.4° in the absence of an inducer (Fig. 2g and h). Thus, although baseline biases in the motor condition are larger than in the perceptual condition, introducing the inducing line adds a fairly consistent 2–3° of additional bias on top of that already present in the pointer-only condition. Hence, the magnitude of the difference in bias between the pointer-only and full Poggendorff conditions was almost identical for perceptual and motor tasks (+4.7° and +4.9°, respectively; *t*_(1,6)_ = 0.16, n.s.), indicating that once the greater baseline landing line bias in motor responses was taken into account, the subsequent effect of adding the Poggendorff inducing line was similar across response modes.

**Fig. 3:**
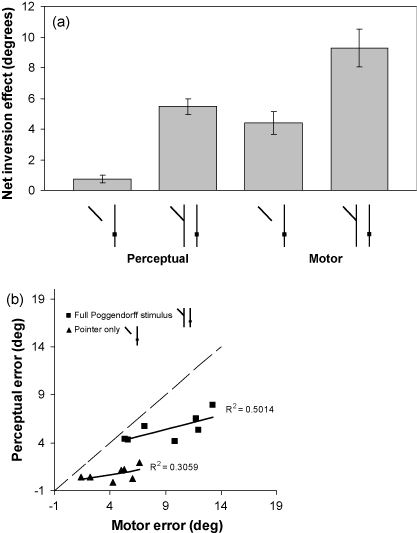
Results of Experiment 1. (a) Mean net inversion effects (bias for inverted stimulus relative to bias to upright stimulus) for each response mode – perceptual or motor – and each stimulus condition – with and without Poggendorff inducing line. The pictorial representations below are for illustrative purposes to clarify the condition, and show the upright configuration of the stimulus; the data in the bar chart depict the difference in bias between these upright configurations and their corresponding inverted configurations (see Fig. 2). Error bars represent S.E.M. A two-factor ANOVA confirmed significant main effects of stimulus type whereby biases were larger with the Poggendorff inducing line than with just the pointer [*F*_(1,8)_ = 227, *p* < 0.0001] and of response mode, whereby biases were larger for motor responses than perceptual response [*F*_(1,8)_ = 6.12, *p* < 0.05]. (b) Net inversion effects for each subject. The diagonal line depicts parity (i.e. equal bias for perceptual and motor responses). The fact that every subject falls below the line shows that motor biases were always greater.

These results are summarised in Fig. 3a (group mean data) and Fig. 3b (individual data). In addition, data (net inversion effects) were entered into a two-factor response mode (perception, action) × stimulus type (full, pointer-only figure) analysis of variance (ANOVA). A significant main effect of response mode confirmed that motor biases were significantly greater than perceptual biases [*F*_(1,6)_ = 33.1, *p* < 0.01], and in addition, that the presence of the inducing line increased biases systematically [the classic Poggendorff effect; *F*_(1,6)_ = 27.1, *p* < 0.01]. This finding was further supported by the absence of any interaction between response mode and stimulus type in the ANOVA [*F*_(1,6)_ = 0.03, n.s.]. A similar analysis of standard deviations revealed that whilst motor responses were more variable than perceptual responses [*F*_(1,6)_ = 100.4, *p* < 0.001], the addition of the inducing line did not affect standard deviations within each response mode [*F*_(1,6)_ = 3.5, n.s.], indicating that the Poggendorff effect systematically biased both responses without influencing their variability (Tibber, Melmoth, & Morgan, 2008).

### 3.2 Pointing kinematics and errors

**Fig. 4:**
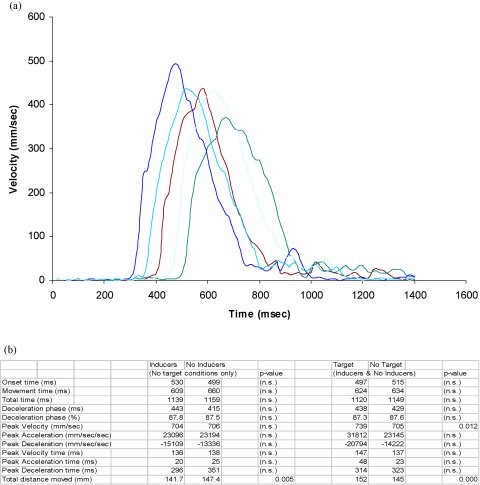
Representative velocity profiles for pointing movements and average values of key kinematic variables, along with *p*-values of paired *t*-tests. For explanation, see the text.

Movement kinematics were compared for pointing movements made to explicit and extrapolated positions on the landing line. Fig. 4 shows representative velocity profiles of pointing movements made under these two conditions to the vertical Poggendorff figure presented with or without the inducing line, along with average values obtained for several measures of reach planning (e.g., movement onset time, peak acceleration, peak velocity) and execution (e.g., movement time, duration of deceleration phase). Paired *t*-tests revealed few significant differences between these measures for any of the comparisons. The single (highly) significant difference common to both conditions was in the total distance that the finger moved down the landing line, which was reduced when subjects pointed to the extrapolated position in the presence of the line responsible for inducing the illusion, rather than when it was absent. In other words, pointing movements to the conventional Poggendorff figure were indistinguishable from normal pointing to a target on the landing line, except for this systematic under-reach.

Indeed, the same was true for the target versus no target conditions, in that subjects moved their finger a shorter total distance to the extrapolated position in the illusory figure compared to when an explicit target was present. In addition, there was a significant reduction in mean peak reaching velocity in the no target versus target conditions (Fig. 4). This finding suggests that the under-reaching bias in the motor extrapolation task was programmed during pointing preparation, since it is well-known that peak reaching velocity increases linearly with estimates of increasing distance available prior to movement onset. Since subjects were required to initiate their pointing response to the Poggendorff illusion as soon as the inducer became visible and there was no difference in their onset or ‘reaction’ times – regardless of whether an actual target was present or not (Fig. 4) – this strongly suggests that the responses resulted from motor programming performed in real-time immediately after the stimulus appeared and, thus, were the product of dorsal stream processing mechanisms.

### 3.3 Experiment 2: increased planning time does not affect the larger motor response bias

In Experiment 1, subjects had only a short period in which to plan their movement and had to generate a new motor program for each pointing response, as the angle of the pointer was varied from trial-to-trial. We wished to discover whether the larger motor bias reported could be eliminated by making the task more predictable, thus potentially engaging ventral stream processes. Using 9 subjects we performed a more stereotyped version of the tasks in which there was no ± 0.5° jitter in the pointer angle and a 3 s preview of each stimulus configuration was given, thus increasing the planning time available for the motor responses. However, we found no differences between this modified procedure and the previous one. Average net inversion effects remained greater for motor (+9.4 ± 1.4° [S.E.M.]) than for perceptual (+7.1 ± 0.9°) responses [*F*_(1,8)_ = 6.14, *p* < 0.05], compared with +9.3° and +5.4° for Experiment 1. Pointer-only conditions again showed that perceptual responses were almost veridical (net inversion effect of +1.6 ± 0.5°) whilst motor responses (net inversion effect of +4.3 ± 0.9°) retained a sizeable landing line bias [*F*_(1,8)_ = 231.6, *p* < 0.001]. There was also no response mode × stimulus type interaction [*F*_(1,8)_ = 0.9, n.s.], showing that additional motor bias came solely from the landing line bias in the pointer-only condition, and that this was independent of the planning time available.

## 4 Experiment 3: similar biases occur for horizontally oriented Poggendorff figures

**Fig. 5:**
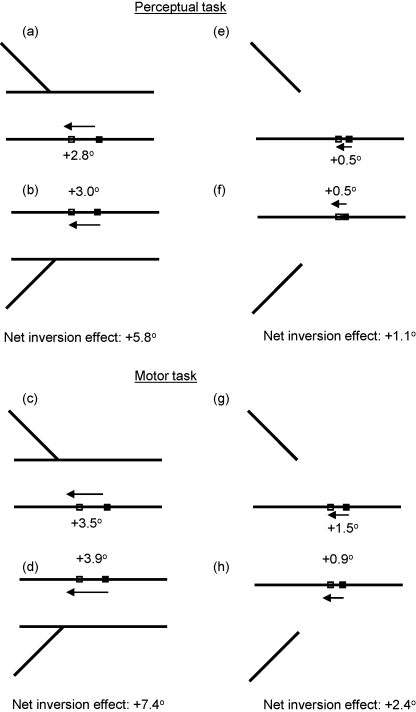
Results of Experiment 3, which used horizontal stimulus configurations. Conventions are the same as in Fig. 2. Note that from the same start positions as Experiments 1 and 2 (the distal end of the pointer) the biased trajectories now fall on the other side of the pointer compared to Fig. 2. Biases demonstrate the same pattern as Experiments 1 and 2, but are generally smaller in magnitude than for the vertical configurations.

Experimental conditions were the same as in Experiment 2, except for the use of a horizontal rather than a vertical configuration (see Fig. 4). The purpose was to see whether part of the motor bias was due to an under-reach in the unknown dimension of the target. Pointing to the extrapolated target location requires that subjects move their fingertip from the start position at the distal end of the oblique pointer along a particular trajectory to an extrapolated position on the landing line. Subjects always knew the approximate direction of the movement (down-right for a top-left start position and upright for a bottom-left start position), but they had to choose the magnitude of this movement. Since the landing line explicitly informs subjects how far they must move their finger horizontally across the screen, their decision centres around the vertical component of the movement vector, so that the motor bias may be thought of as an error in placing their finger in the (unknown) vertical dimension. Specifically, Fig. 2 shows that they do not move far enough in the vertical dimension from their start position. Thus, the motor bias can be considered an “under-reach” in this unknown dimension of movement. However, an alternative conception is that in an extrapolation task, the vertical component of the movement is in general decreased relative to the horizontal. To distinguish between these possibilities, we presented stimuli in a horizontal configuration. This kept finger start positions and pointing directions the same, but the unknown dimension of movement was now the horizontal, since the landing line now explicitly indicated how far subjects must move their finger in the vertical domain. As shown in Fig. 5, the results were consistent with the first hypothesis: subjects now under-reached in the (unknown) horizontal dimension. As before, whilst both the perception and action systems were subject to the full Poggendorff illusion, with mean net inversion effects being significantly greater for motor than perceptual responses [*F*_(1,8)_ = 7.97, *p* < 0.05], the landing line bias in the pointer-only conditions was predominantly seen only in motor responses.

**Fig. 6:**
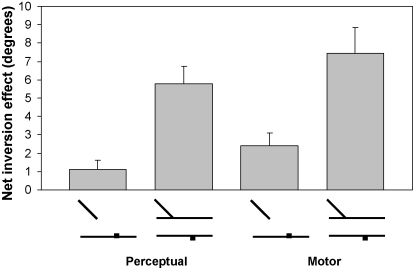
Results of Experiment 3, showing mean net inversion effects (bias for inverted stimulus relative to bias to upright stimulus) for each response mode – perceptual or motor – and each stimulus condition, i.e. with and without Poggendorff inducing line, in the horizontal configuration. The pictorial representations below are for illustrative purposes to clarify the condition, and show the upright configuration of the stimulus; the data in the bar chart depict the difference in bias between these upright configurations and their corresponding inverted configurations (see Fig. 4). Error bars represent S.E.M. Magnitude of biases is smaller than for the vertical configurations (Fig. 3), but a two-factor ANOVA confirmed the same pattern of results: significant main effects of stimulus type, whereby biases were larger with the Poggendorff inducing line than with just the pointer [*F*_(1,8)_ = 48.1, *p* < 0.001] and of response mode, whereby biases were larger for motor responses than perceptual response [*F*_(1,8)_ = 7.97, *p* < 0.05].

Fig. 6 shows mean net inversion effects for each condition. The pattern of results is exactly the same as for Experiments 1 and 2, although the effects are generally weaker with these horizontal stimuli than for the corresponding vertical conditions of Experiment 2, which is consistent with previous findings on perceptual responses to the Poggendorff illusion. Net inversion effects for the full Poggendorff stimulus were +5.8° and +7.4° for the perceptual and motor biases, respectively, whilst for the landing line bias in the pointer-only conditions net inversion effect were +1.1° and +2.4°, respectively. A two-factor (response mode, inducers) ANOVA again confirmed significant effects of the Poggendorff inducing line – with greater biases when the inducing line was present compared to pointer-only conditions [*F*_(1,8)_ = 48.1, *p* < 0.001] – and once again of response mode, with motor bias being greater than perceptual bias [*F*_(1,8)_ = 7.97, *p* < 0.05]. The idea that the effect of the inducer and of the response mode are additive factors was further supported by the absence of any interaction between response mode and stimulus type in the ANOVA [*F*_(1,8)_ = 0.12, n.s.].

### 4.1 Experiment 4: eliminating the motor landing line bias

In the final experiment, conditions were the same as in Experiments 2 and 3, except that the landing line was rotated 90° with respect to the inducer. The data presented so far suggest that two independent, but additive, factors affect motor responses: the acute angle of the traditional Poggendorff figure (which also affects perception), and a second bias which is unique to the motor condition (an effect we shall call the landing line bias as it persists in the absence of an inducer). These effects are mediated by different parts of the full Poggendorff stimulus, but, for the configurations used so far (with parallel Poggendorff lines) they behave additively in the same angular direction, producing a large motor bias. We therefore hypothesised that by rotating the landing line so that it is orthogonal to the inducing line these two independent effects upon motor responses could be set in opposition to each other, behaving subtractively. Thus, the “under-reach” along the landing line seen in motor responses, would produce a trajectory bias *opposite* to the trajectory bias caused by expansion of the acute Poggendorff angle. Since the landing line bias had little effect upon perceptual responses, we did not expect its rotation to impact upon perceptual responses, i.e. they will be affected by the acute Poggendorff angle alone, just as before. Therefore, our prediction was that motor biases would become *smaller* than the perceptual biases when the two separate effects were set in opposition.

**Fig. 7:**
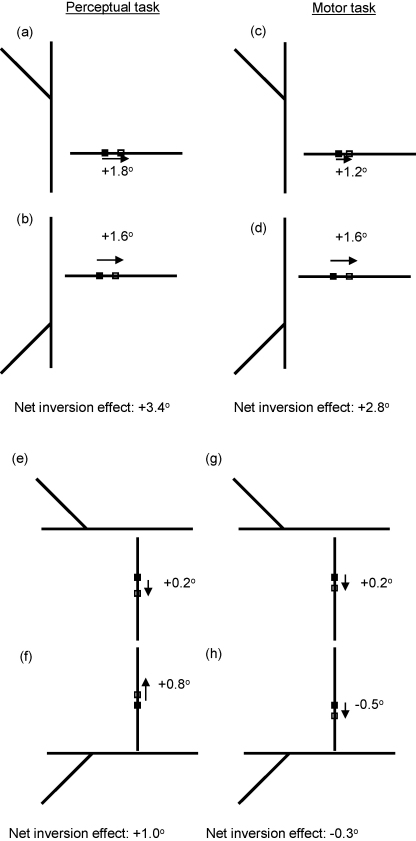
Results and pictorial representations for Experiment 4, which measured illusory biases with the “opposing” stimuli: i.e. the inducing and landing lines are orthogonal so that the respective biases each line mediates are now in opposition. (a–d) Configurations for vertical inducer with horizontal landing line; (e–h) configurations for horizontal inducer with vertical landing line. Note that motor response biases are now almost completely nulled by the opposing effects.

**Fig. 8:**
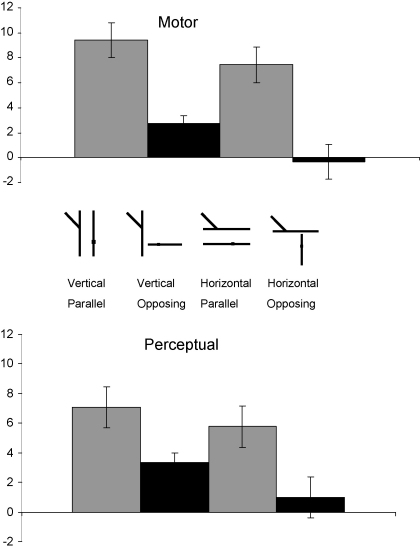
The figure compares net inversion effects of Experiment 4 with those in comparable conditions of previous experiments (Experiments 2 and 3). Error bars represent S.E.M. For the vertical inducer conditions (which illicit the strongest acute angle Poggendorff effect) the opposing landing line bias (which is weakest for horizontal landing lines) diminished but did not overpower the Poggendorff effect for motor responses. However, in the horizontal inducer condition the opposing bias from the orthogonal landing line fully overpowers the Poggendorff effect, completely nulling motor response bias. A similar, but weaker, pattern of results occurs for perceptual responses.

As predicted (Figs. 7 and 8), the motor bias was reduced in the orthogonal landing line condition relative to the parallel landing line condition. However, this reduction in bias was found for both perceptual and motor conditions. We realised *post-facto* that this reduction of the perceptual effect is expected on the basis of a previous report by Weintraub and Krantz (1971) which demonstrates that the Poggendorff effect is weaker without a second parallel. Nonetheless, our prediction from the additive factor hypothesis is supported by the significantly greater effect of rotating the landing line upon the motor bias (*F*_(1,8)_ = 5.43, *p* < 0.05). However, the lack of a significantly greater perceptual effect with the orthogonal landing line means that the Poggendorff bias and the putative motor undereaching cannot be treated as linearly additive factors. Instead, a more complex interaction must exist between the two biases, a relationship that will be explored further in ongoing experiments.

## 5 Discussion

Our initial aim was to determine whether rapid pointing responses would be affected by the Poggendorff illusion. We confirmed that they were. Although motor response biases to other visual illusions have been used to argue against the duplex theory of vision, our result does not necessarily contradict the two streams hypothesis for perception and action. Milner and Dyde (2003) proposed that if an illusion manifests itself early in the visual system then both the dorsal and ventral streams will inherit the bias. Such a shared inheritance could explain an equal bias of both motor and perceptual responses. Since the Poggendorff illusion is presumed to manifest itself early, perhaps due to expansion of the acute angle due to lateral inhibition of orientation detectors (Blakemore, Carpenter, & Georgeson, 1970); ‘bowing’ of the transversal pointer (Wenderoth, 1980), or blurring of second stage filters in V2 (Morgan, 1999), our results do not contradict Milner and Dyde's proposal. Thus, the common bias of the Poggendorff effect upon perception and action, which added a consistent 2–3^o^ of bias on top of whatever landing line bias was already present in the pointer-only condition (Fig. 2), is consistent with the notion that angular illusions of this type arise before the division into dorsal and ventral processing streams, possibly in V1 or V2.

Another possibility, which is also consistent with our findings, is that all motor responses will show the same illusory biases as perceptual judgements, subject to procedural differences. Franz, Hesse, and Collath (2008) argued recently that recorded motor biases may often be smaller than recorded perceptual biases because motor responses can be modified on-line by visual feedback, thereby reducing the magnitude of the initially programmed response bias. Franz's hypothesis would predict a strong illusion in our pointing conditions since unlike size illusions where visual feedback about the accuracy of grip size can be obtained in flight, the observer in our Poggendorff task has no explicit target to correct their motor response during the trajectory.

There is little experimental precedent for a motor response bias being *greater* than the corresponding perceptual response bias to a given stimuli (e.g., Bruno, Bernardis, & Gentilucci, 2008) other than when deliberately manipulated (e.g., Dyde & Milner, 2002) or when the tasks may be mis-matched (Franz et al., 2001). Interestingly though, one example of a greater motor bias from Bruno and Bernardis (2003), occurred when mental extrapolation was required, as is also required in the Poggendorff task. We found larger motor response biases to the full Poggendorff stimulus because of the presence of a motor-specific bias to the landing line alone – despite near-veridical perceptual responses – which added to the shared inherited bias of the Poggendorff inducing line. The motor bias can be thought of as a consistent ‘under-reach’ in the unknown dimension of hand movement. This may not represent an illusion, but could simply reflect a deliberate strategy. Accurate reaching or pointing movements are presumed to require on-line corrections to an initial motor programme (Glover & Dixon, 2001), which predominantly occur late in the movement (Grant, Melmoth, Morgan, & Finlay, 2007; Melmoth & Grant, 2006; Melmoth, Storoni, Todd, Finlay, & Grant, 2007). Under sub-optimal viewing conditions subjects adopt a conservative strategy for prehension movements, which includes under-reaching (Bradshaw & Watt, 2002; Elliott, Carson, Goodman, & Chua, 1991; Elliott & Lee, 1995; Grant et al., 2007; Melmoth & Grant, 2006; Melmoth et al., 2007; Watt & Bradshaw, 2000) and subsequent correction. In our current task, there is a region of uncertainty within which the true extrapolated target position is located. A conceivably efficient evolved motor strategy for reaching would be to take the effector (in this case the finger) to the nearest boundary of the region of uncertainty and then make any additional corrections if necessary. Since, in our experiments, no additional feedback is available by which to make any further correction, no such adjustments are made and the initial under-reach remains. One difference between the response modes was that on half of the motor response trials the true location of the extrapolated intersection was explicitly shown, with subjects required to point to it. This was necessary due to the recording set-up, which required that we measure baseline performance to subtract the discrepancy between the location of the reflective marker (placed on the finger *nail*) and the finger*tip* placed on the screen. However, if anything, this would be expected to *improve* motor response accuracy, relative to perceptual responses, so cannot account for the greater motor bias.

Another difference between response modes was the duration over which they took place since ballistic pointing movements are, by their nature, quicker than an iterative adjustment task. Pointing movements in our experiment took approximately 1200 ms to complete (500 ms to initiate movement and 700 ms to execute), whilst perceptual responses were unconstrained and took much longer. Accordingly, response time for the perceptual task could have been constrained to match the natural durations of pointing movements—for example, via a “one-shot” perceptual task, or a 2AFC. However, Franz (2003) reports that using 2AFC method-of-constant-stimuli to test perceptual biases to illusions with time constraints down to 800 ms produced the same results as using time-unconstrained method of adjustment. Likewise, if the Poggendorff illusion manifests early (e.g., in V1) then it is unclear what theoretical framework exists for supposing that allowing multiple eye movements in our perceptual task reduced the strength of the illusion. Nevertheless, we are currently investigating time-constrained perceptual responses since the possibility remains that the additional bias found in the motor responses is not specifically inherent to the motor (dorsal) system, but rather reflects a difference in the speed/duration of response and that by suitably constraining perceptual response conditions a similar bias could become manifest. A confound of this is that eye movements are, in themselves, a motor response and we have preliminary evidence that they too are subject to the motor-specific landing line bias. If these saccadic movements are used as the basis of a “perceptual” judgement, then we may indeed expect them to affect perceptual response biases.

Future experiments will also test motor responses when the motor response does not have the same vector as the extrapolated line, i.e. when the reaching movement does not follow the trajectory of the pointer. For example, when pointing direction is orthogonal to the extrapolated line, if the concept of an under-reach holds angular bias would occur in the opposite direction to those reported here.

That the additional motor landing line bias is separate from any bias arising from the Poggendorff illusion is apparent both from the fact that it occurs in the absence of the inducing line, and that when the landing line and inducing line are set orthogonally the two biases oppose each other, reducing overall bias. A remaining question is why *perceptual* biases with these ‘opposing’ configurations in Experiment 4, were reduced compared to Experiment 1. The finding is not unprecedented. For example, Weintraub and Krantz (1971) found that the Poggendorff effect was weaker without a second parallel. It is also known that the full Poggendorff stimulus consists of numerous compounded visual illusions such as the vertical–horizontal extent, Wundt, Zehender and Obonai illusions to mention just a few (e.g., Day, 1973; Goldstein & Weintraub, 1972; Hotopf, 1981; Hotopf & Hibberd, 1989; Pressey & Swinney, 1969; Tibber et al., 2008; Weintraub & Krantz, 1971). These interacting effects are mediated by different component elements of the full Poggendorff stimulus so it is no surprise that the net perceptual bias is affected by changing the stimulus configuration. It should also be remembered that this reduction was significantly smaller than for the corresponding motor responses.

## 6 Conclusion

We have found that the acute angle version of the Poggendorff illusion affects perceptual (adjustment) and motor (rapid pointing) tasks equally, supporting the notion of a shared inheritance of this bias. However, in addition, there is a motor effect without a perceptual counterpart, when the acute angle is absent, resulting in larger total motor bias to the full Poggendorff stimulus. The motor effect appears as an under-reach in the unknown component of the pointing vector, which could be explained by a strategy to reduce effort. Finally, the dynamics of pointing to an extrapolated target position are the same as those for pointing at an explicit target, once the difference in path lengths are accounted for.
